# Ethyl 4-(4-chloro­phen­yl)-2-methyl-5-oxo-5,6,7,8-tetra­hydro­quinoline-3-carboxyl­ate

**DOI:** 10.1107/S1600536813016541

**Published:** 2013-06-19

**Authors:** Ke Wang, Weike Wang, Yifeng Wang, Danqian Xu

**Affiliations:** a Catalytic Hydrogenation Research Center, Zhejiang University of Technology, Hangzhou 310014, People’s Republic of China; b Zhejiang Huanke Environment Consultancy Co.,Ltd, No. 111 Tianmushan Road, Hangzhou 310007, People’s Republic of China

## Abstract

In the title compound, C_19_H_18_ClNO_3_, the non-aromatic part of the fused ring system adopts an envelope conformation with the central methyl­ene C atom as the flap. The dihedral angle between the pyridine and benzene rings is 56.98 (3)°. In the crystal, mol­ecules are linked into double layers parallel to (100) by a network of weak C—H⋯O inter­actions.

## Related literature

For the synthetic procedure, see: Fang *et al.* (2007 ▶); Mirza-Aghayan *et al.* (2012 ▶). For a related structure, see: Sicheri *et al.* (1992 ▶).
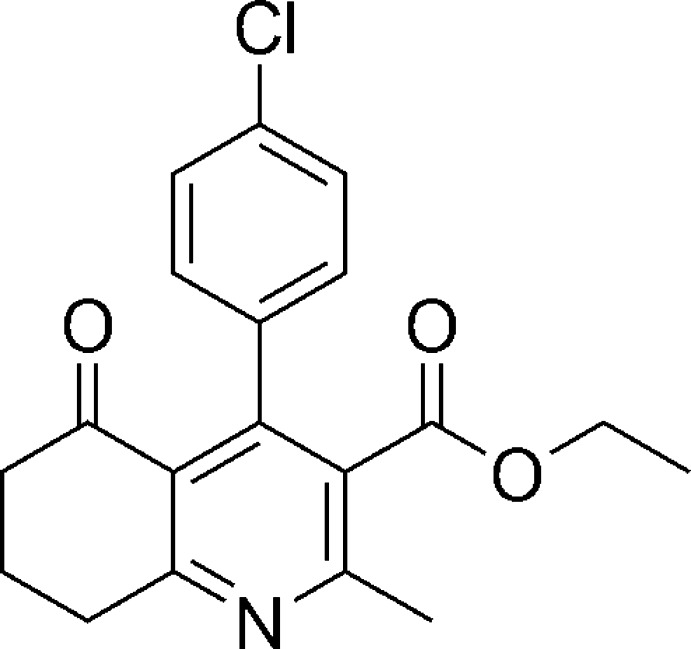


## Experimental

### 

#### Crystal data

C_19_H_18_ClNO_3_
*M* *_r_* = 343.79
Monoclinic, 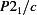

*a* = 12.5736 (7) Å
*b* = 8.3815 (4) Å
*c* = 17.4945 (8) Å
β = 112.151 (2)°
*V* = 1707.59 (15) Å^3^
*Z* = 4
Mo *K*α radiation
μ = 0.24 mm^−1^
*T* = 296 K
0.49 × 0.42 × 0.30 mm

#### Data collection

Rigaku R-AXIS RAPID diffractometer
Absorption correction: multi-scan (*ABSCOR*; Higashi, 1995 ▶) *T*_min_ = 0.891, *T*_max_ = 0.932
15658 measured reflections
3860 independent reflections
2725 reflections with *I* > 2σ(*I*)
*R*_int_ = 0.053

#### Refinement

*R*[*F* ^2^ > 2σ(*F* ^2^)] = 0.050
*wR*(*F* ^2^) = 0.128
*S* = 1.00
3860 reflections
220 parameters
H-atom parameters constrained
Δρ_max_ = 0.28 e Å^−3^
Δρ_min_ = −0.26 e Å^−3^

### 

Data collection: *PROCESS-AUTO* (Rigaku, 2006 ▶); cell refinement: *PROCESS-AUTO*; data reduction: *CrystalStructure* (Rigaku, 2007 ▶); program(s) used to solve structure: *SHELXS97* (Sheldrick, 2008 ▶); program(s) used to refine structure: *SHELXL97* (Sheldrick, 2008 ▶); molecular graphics: *ORTEP-3 for Windows* (Farrugia, 2012 ▶); software used to prepare material for publication: *WinGX* (Farrugia, 2012 ▶).

## Supplementary Material

Crystal structure: contains datablock(s) global, I. DOI: 10.1107/S1600536813016541/fy2095sup1.cif

Structure factors: contains datablock(s) I. DOI: 10.1107/S1600536813016541/fy2095Isup2.hkl

Supplementary material file. DOI: 10.1107/S1600536813016541/fy2095Isup3.cml

Additional supplementary materials:  crystallographic information; 3D view; checkCIF report

## Figures and Tables

**Table 1:** Hydrogen-bond geometry (Å, °)

*D*—H⋯*A*	*D*—H	H⋯*A*	*D*⋯*A*	*D*—H⋯*A*
C6—H6*B*⋯O3^i^	0.97	2.67	3.454 (3)	139
C11—H11⋯O1^ii^	0.93	2.45	3.357 (2)	164
C12—H12⋯O3^iii^	0.93	2.70	3.569 (2)	156

Symmetry codes: (i) 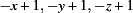; (ii) 
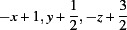
; (iii) 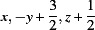.

**Fig. 1.**
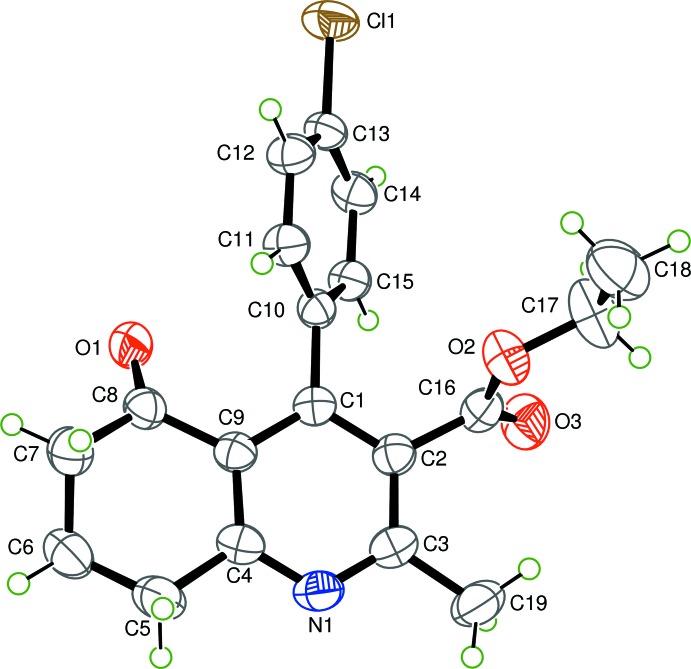
Molecular structure of the title compound. Displacement ellipsoids are drawn at the 40% probability level.

**Fig. 2.**
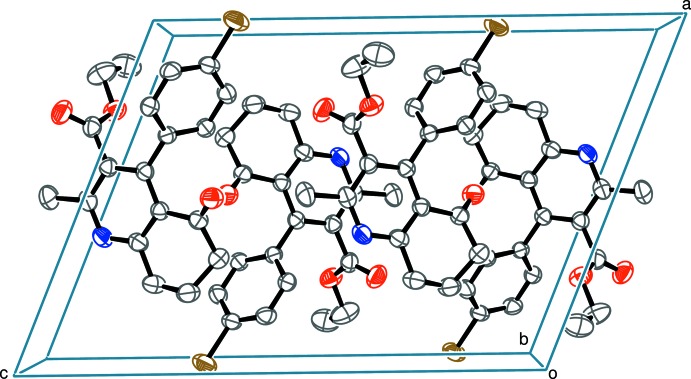
Crystal packing of the title compound. H atoms have been omitted for clarity.

## supplementary crystallographic information

### Comment

The pyridine nucleus is of substantial significance as it is the key component in a variety of bioactive compounds, both naturally occurring and synthetic. The oxidative aromatization of 1,4-dihydropyridines is a very convenient approach to the synthesis of highly substituted pyridines (Fang *et al.*, 2007; Mirza-Aghayan *et al.*, 2012). In this article, the title compound was synthesized from the oxidation of the corresponding 1,4-dihydropyridine, and the crystal structure of it is described (Fig. 1). The non-aromatic part of the fused ring is non-planar and adopts an envelope conformation. The α-carbon atom of the carbonyl lies on the same side of the fused ring with the ethyl group, whereas the β-carbon atom of the carbonyl was oriented in opposite direction. The dihedral angle between the pyridine ring and the benzene ring is 56.98 (3)°. In the crystal,molecules are linked by weak C—H···O interactions.

### Experimental

The mixture of 4-chlorobenzaldehyde (1 mmol), ethylacetoacetate (1 mmol) and 3-aminocyclohex-2-enone (1 mmol) was stirred at at 343 K for 3 h (monitored by TLC). Then the mixture was purified by flash column chromatography (silica gel, Hex/AcOEt, *v*/*v*, 3:1) giving the 1,4-dihydropyridine compound. The 1,4-dihydropyridine compound was further oxidized by H_2_O_2_ (2.0 equiv.) in the presence of the PEG1000-BMImI complex catalyst (50 mol%) to afford the title compound. The pure pruduct is obtained through recrystallation, and single crystals were obtained by slow evaporation of a dichloromethane/n-hexane (1:1 *v*/*v*) solution at room temperature.

### Refinement

Methyl H atoms were placed in calculated positions with C—H = 0.96 (1) Å and the torsion was refined to fit the electron density with *U*_iso_(H) = 1.5*U*_eq_(C). Other H atoms were placed in calculated positions and treated as riding atoms: C—H = 0.97 (1) Å (sp^3^) and C—H = 0.93 Å (aromatic) with *U*_iso_(H) = 1.2*U*_eq_(C).

### Crystal data

**Table:**

C_19_H_18_ClNO_3_	*F*(000) = 720
*M**_r_* = 343.79	*D*_x_ = 1.337 Mg m^−^^3^
Monoclinic, *P*2_1_/*c*	Mo *K*α radiation, λ = 0.71073 Å
Hall symbol: -P 2ybc	Cell parameters from 10595 reflections
*a* = 12.5736 (7) Å	θ = 3.1–27.4°
*b* = 8.3815 (4) Å	µ = 0.24 mm^−^^1^
*c* = 17.4945 (8) Å	*T* = 296 K
β = 112.151 (2)°	Chunk, yellow
*V* = 1707.59 (15) Å^3^	0.49 × 0.42 × 0.30 mm
*Z* = 4	

### Data collection

**Table:**

Rigaku R-AXIS RAPID diffractometer	3860 independent reflections
Radiation source: rotating anode	2725 reflections with *I* > 2σ(*I*)
Graphite monochromator	*R*_int_ = 0.053
Detector resolution: 10.00 pixels mm^-1^	θ_max_ = 27.5°, θ_min_ = 3.0°
ω scans	*h* = −16→16
Absorption correction: multi-scan (*ABSCOR*; Higashi, 1995)	*k* = −10→10
*T*_min_ = 0.891, *T*_max_ = 0.932	*l* = −18→22
15658 measured reflections	

### Refinement

**Table:**

Refinement on *F*^2^	Secondary atom site location: difference Fourier map
Least-squares matrix: full	Hydrogen site location: inferred from neighbouring sites
*R*[*F*^2^ > 2σ(*F*^2^)] = 0.050	H-atom parameters constrained
*wR*(*F*^2^) = 0.128	*w* = 1/[σ^2^(*F*_o_^2^) + (0.0515*P*)^2^ + 0.6492*P*] where *P* = (*F*_o_^2^ + 2*F*_c_^2^)/3
*S* = 1.00	(Δ/σ)_max_ = 0.001
3860 reflections	Δρ_max_ = 0.28 e Å^−^^3^
220 parameters	Δρ_min_ = −0.26 e Å^−^^3^
0 restraints	Extinction correction: *SHELXL97* (Sheldrick, 2008), Fc^*^=kFc[1+0.001xFc^2^λ^3^/sin(2θ)]^-1/4^
Primary atom site location: structure-invariant direct methods	Extinction coefficient: 0.043 (3)

### Special details

**Table:**

Geometry. All e.s.d.'s (except the e.s.d. in the dihedral angle between two l.s. planes) are estimated using the full covariance matrix. The cell e.s.d.'s are taken into account individually in the estimation of e.s.d.'s in distances, angles and torsion angles; correlations between e.s.d.'s in cell parameters are only used when they are defined by crystal symmetry. An approximate (isotropic) treatment of cell e.s.d.'s is used for estimating e.s.d.'s involving l.s. planes.
Refinement. Refinement of *F*^2^ against ALL reflections. The weighted *R*-factor *wR* and goodness of fit *S* are based on *F*^2^, conventional *R*-factors *R* are based on *F*, with *F* set to zero for negative *F*^2^. The threshold expression of *F*^2^ > σ(*F*^2^) is used only for calculating *R*-factors(gt) *etc*. and is not relevant to the choice of reflections for refinement. *R*-factors based on *F*^2^ are statistically about twice as large as those based on *F*, and *R*- factors based on ALL data will be even larger.

### Fractional atomic coordinates and isotropic or equivalent isotropic displacement parameters (Å^2^)

**Table:**

	*x*	*y*	*z*	*U*_iso_*/*U*_eq_	
C1	0.42545 (16)	0.6493 (2)	0.58924 (11)	0.0375 (4)	
C2	0.41107 (17)	0.7151 (2)	0.51265 (11)	0.0413 (4)	
C3	0.50712 (19)	0.7626 (2)	0.49622 (12)	0.0473 (5)	
C4	0.62896 (17)	0.6962 (2)	0.62798 (12)	0.0436 (4)	
C5	0.75005 (18)	0.7044 (3)	0.69010 (14)	0.0580 (6)	
H5A	0.7673	0.8142	0.7080	0.070*	
H5B	0.8018	0.6734	0.6633	0.070*	
C6	0.77367 (19)	0.6007 (3)	0.76517 (14)	0.0581 (6)	
H6A	0.8455	0.6329	0.8081	0.070*	
H6B	0.7811	0.4905	0.7511	0.070*	
C7	0.67692 (18)	0.6145 (3)	0.79737 (12)	0.0539 (5)	
H7A	0.6935	0.5475	0.8457	0.065*	
H7B	0.6707	0.7240	0.8132	0.065*	
C8	0.56577 (17)	0.5633 (2)	0.73116 (12)	0.0426 (4)	
C9	0.53856 (16)	0.6381 (2)	0.64827 (11)	0.0388 (4)	
C10	0.32257 (15)	0.6031 (2)	0.60723 (11)	0.0368 (4)	
C11	0.30200 (17)	0.6742 (2)	0.67217 (11)	0.0426 (4)	
H11	0.3545	0.7475	0.7056	0.051*	
C12	0.20518 (18)	0.6381 (2)	0.68790 (12)	0.0466 (5)	
H12	0.1922	0.6862	0.7315	0.056*	
C13	0.12768 (16)	0.5288 (2)	0.63758 (12)	0.0434 (4)	
C14	0.14541 (17)	0.4562 (2)	0.57310 (12)	0.0461 (5)	
H14	0.0925	0.3829	0.5400	0.055*	
C15	0.24351 (16)	0.4933 (2)	0.55776 (11)	0.0426 (4)	
H15	0.2562	0.4445	0.5142	0.051*	
C16	0.29397 (18)	0.7489 (2)	0.44907 (11)	0.0454 (5)	
C17	0.1340 (2)	0.9227 (3)	0.41726 (18)	0.0778 (8)	
H17A	0.0757	0.8430	0.4117	0.093*	
H17B	0.1365	0.9418	0.3633	0.093*	
C18	0.1069 (2)	1.0718 (3)	0.4508 (2)	0.0846 (8)	
H18A	0.1020	1.0508	0.5033	0.127*	
H18B	0.0348	1.1129	0.4134	0.127*	
H18C	0.1663	1.1488	0.4576	0.127*	
C19	0.4969 (2)	0.8326 (3)	0.41431 (14)	0.0654 (6)	
H19A	0.5631	0.8971	0.4216	0.098*	
H19B	0.4290	0.8974	0.3930	0.098*	
H19C	0.4920	0.7480	0.3761	0.098*	
Cl1	0.00542 (5)	0.48132 (7)	0.65772 (4)	0.0647 (2)	
N1	0.61354 (15)	0.7536 (2)	0.55298 (11)	0.0497 (4)	
O1	0.50420 (13)	0.46307 (17)	0.74340 (9)	0.0545 (4)	
O2	0.24504 (13)	0.86760 (17)	0.47419 (9)	0.0560 (4)	
O3	0.25193 (14)	0.68423 (19)	0.38335 (9)	0.0642 (5)	

### Atomic displacement parameters (Å^2^)

**Table:**

	*U*^11^	*U*^22^	*U*^33^	*U*^12^	*U*^13^	*U*^23^
C1	0.0394 (10)	0.0356 (9)	0.0393 (10)	−0.0022 (7)	0.0169 (8)	−0.0045 (7)
C2	0.0432 (11)	0.0426 (10)	0.0390 (10)	−0.0019 (8)	0.0166 (8)	−0.0033 (7)
C3	0.0537 (13)	0.0487 (11)	0.0459 (11)	−0.0031 (9)	0.0262 (10)	−0.0020 (8)
C4	0.0394 (11)	0.0446 (10)	0.0500 (11)	−0.0014 (8)	0.0205 (9)	−0.0058 (8)
C5	0.0382 (12)	0.0722 (14)	0.0639 (14)	−0.0048 (10)	0.0196 (10)	−0.0048 (11)
C6	0.0400 (12)	0.0701 (14)	0.0582 (13)	0.0018 (10)	0.0117 (10)	−0.0068 (10)
C7	0.0464 (12)	0.0677 (13)	0.0432 (11)	−0.0007 (10)	0.0117 (9)	−0.0050 (9)
C8	0.0390 (10)	0.0466 (10)	0.0423 (10)	0.0047 (8)	0.0155 (8)	−0.0018 (8)
C9	0.0383 (10)	0.0395 (9)	0.0397 (10)	−0.0003 (7)	0.0161 (8)	−0.0045 (7)
C10	0.0337 (9)	0.0383 (9)	0.0365 (9)	−0.0006 (7)	0.0111 (7)	0.0014 (7)
C11	0.0406 (11)	0.0461 (10)	0.0408 (10)	−0.0086 (8)	0.0149 (8)	−0.0091 (8)
C12	0.0471 (12)	0.0515 (11)	0.0461 (11)	−0.0019 (8)	0.0231 (9)	−0.0066 (8)
C13	0.0330 (10)	0.0481 (10)	0.0501 (11)	−0.0007 (8)	0.0168 (8)	0.0036 (8)
C14	0.0376 (11)	0.0476 (10)	0.0492 (11)	−0.0089 (8)	0.0117 (9)	−0.0075 (8)
C15	0.0410 (11)	0.0470 (10)	0.0394 (10)	−0.0026 (8)	0.0148 (8)	−0.0067 (7)
C16	0.0505 (12)	0.0481 (10)	0.0382 (10)	−0.0041 (9)	0.0173 (9)	0.0018 (8)
C17	0.0601 (16)	0.0666 (15)	0.0816 (18)	0.0133 (12)	−0.0019 (13)	−0.0026 (13)
C18	0.0680 (18)	0.0735 (17)	0.112 (2)	0.0162 (13)	0.0335 (17)	0.0057 (16)
C19	0.0703 (16)	0.0826 (16)	0.0528 (13)	−0.0089 (12)	0.0341 (12)	0.0065 (11)
Cl1	0.0444 (3)	0.0791 (4)	0.0791 (4)	−0.0094 (3)	0.0330 (3)	−0.0020 (3)
N1	0.0463 (10)	0.0581 (10)	0.0523 (10)	−0.0038 (8)	0.0271 (8)	−0.0016 (8)
O1	0.0472 (9)	0.0600 (9)	0.0539 (9)	−0.0002 (7)	0.0163 (7)	0.0149 (7)
O2	0.0498 (9)	0.0548 (8)	0.0536 (9)	0.0088 (6)	0.0084 (7)	−0.0054 (6)
O3	0.0649 (11)	0.0769 (10)	0.0418 (8)	0.0010 (8)	0.0100 (7)	−0.0117 (7)

### Geometric parameters (Å, º)

**Table:**

C1—C2	1.396 (3)	C10—C11	1.391 (3)
C1—C9	1.411 (3)	C11—C12	1.379 (3)
C1—C10	1.493 (3)	C11—H11	0.9300
C2—C3	1.400 (3)	C12—C13	1.385 (3)
C2—C16	1.500 (3)	C12—H12	0.9300
C3—N1	1.334 (3)	C13—C14	1.372 (3)
C3—C19	1.509 (3)	C13—Cl1	1.748 (2)
C4—N1	1.341 (3)	C14—C15	1.393 (3)
C4—C9	1.400 (3)	C14—H14	0.9300
C4—C5	1.502 (3)	C15—H15	0.9300
C5—C6	1.508 (3)	C16—O3	1.199 (2)
C5—H5A	0.9700	C16—O2	1.329 (2)
C5—H5B	0.9700	C17—O2	1.451 (3)
C6—C7	1.525 (3)	C17—C18	1.474 (4)
C6—H6A	0.9700	C17—H17A	0.9700
C6—H6B	0.9700	C17—H17B	0.9700
C7—C8	1.503 (3)	C18—H18A	0.9600
C7—H7A	0.9700	C18—H18B	0.9600
C7—H7B	0.9700	C18—H18C	0.9600
C8—O1	1.215 (2)	C19—H19A	0.9600
C8—C9	1.496 (3)	C19—H19B	0.9600
C10—C15	1.391 (2)	C19—H19C	0.9600
			
C2—C1—C9	117.32 (17)	C12—C11—C10	121.26 (17)
C2—C1—C10	119.73 (16)	C12—C11—H11	119.4
C9—C1—C10	122.86 (16)	C10—C11—H11	119.4
C1—C2—C3	119.96 (18)	C11—C12—C13	118.74 (18)
C1—C2—C16	121.46 (17)	C11—C12—H12	120.6
C3—C2—C16	118.39 (17)	C13—C12—H12	120.6
N1—C3—C2	122.12 (18)	C14—C13—C12	121.52 (18)
N1—C3—C19	115.56 (19)	C14—C13—Cl1	119.53 (15)
C2—C3—C19	122.3 (2)	C12—C13—Cl1	118.95 (15)
N1—C4—C9	122.76 (18)	C13—C14—C15	119.31 (17)
N1—C4—C5	115.12 (18)	C13—C14—H14	120.3
C9—C4—C5	122.07 (18)	C15—C14—H14	120.3
C4—C5—C6	114.62 (18)	C10—C15—C14	120.33 (17)
C4—C5—H5A	108.6	C10—C15—H15	119.8
C6—C5—H5A	108.6	C14—C15—H15	119.8
C4—C5—H5B	108.6	O3—C16—O2	124.34 (19)
C6—C5—H5B	108.6	O3—C16—C2	125.48 (19)
H5A—C5—H5B	107.6	O2—C16—C2	110.09 (16)
C5—C6—C7	110.81 (18)	O2—C17—C18	107.7 (2)
C5—C6—H6A	109.5	O2—C17—H17A	110.2
C7—C6—H6A	109.5	C18—C17—H17A	110.2
C5—C6—H6B	109.5	O2—C17—H17B	110.2
C7—C6—H6B	109.5	C18—C17—H17B	110.2
H6A—C6—H6B	108.1	H17A—C17—H17B	108.5
C8—C7—C6	109.46 (17)	C17—C18—H18A	109.5
C8—C7—H7A	109.8	C17—C18—H18B	109.5
C6—C7—H7A	109.8	H18A—C18—H18B	109.5
C8—C7—H7B	109.8	C17—C18—H18C	109.5
C6—C7—H7B	109.8	H18A—C18—H18C	109.5
H7A—C7—H7B	108.2	H18B—C18—H18C	109.5
O1—C8—C9	122.17 (17)	C3—C19—H19A	109.5
O1—C8—C7	122.06 (18)	C3—C19—H19B	109.5
C9—C8—C7	115.71 (17)	H19A—C19—H19B	109.5
C4—C9—C1	118.81 (17)	C3—C19—H19C	109.5
C4—C9—C8	118.73 (17)	H19A—C19—H19C	109.5
C1—C9—C8	122.44 (17)	H19B—C19—H19C	109.5
C15—C10—C11	118.84 (17)	C3—N1—C4	118.87 (17)
C15—C10—C1	120.88 (16)	C16—O2—C17	117.20 (17)
C11—C10—C1	120.24 (16)		

### Hydrogen-bond geometry (Å, º)

**Table:**

*D*—H···*A*	*D*—H	H···*A*	*D*···*A*	*D*—H···*A*
C6—H6*B*···O3^i^	0.97	2.67	3.454 (3)	139
C11—H11···O1^ii^	0.93	2.45	3.357 (2)	164
C12—H12···O3^iii^	0.93	2.70	3.569 (2)	156

Symmetry codes: (i) −*x*+1, −*y*+1, −*z*+1; (ii) −*x*+1, *y*+1/2, −*z*+3/2; (iii) *x*, −*y*+3/2, *z*+1/2.
